# Morphology, Distribution and Sexual Dimorphism of Sensilla in the Tea Leaf Beetle *Basilepta melanopus* (Coleoptera: Eumolpidae)

**DOI:** 10.3390/biology15191685

**Published:** 2026-09-22

**Authors:** Dingran Zhang, Huan Qiu, Yuetong Guan, Wenzheng Tang, Rongrong Tan, Xun Chen, Hongjuan Wang, Danjuan Huang, Lijin An, Lin Du, Fen Zhu, Long Jiao, Yingxin Mao

**Affiliations:** 1 Key Laboratory of Tea Resources Comprehensive Utilization, Ministry of Agriculture and Rural Affairs, Institute of Fruit and Tea, Hubei Academy of Agricultural Sciences, Wuhan 430064, China; 13523738233@163.com (D.Z.); huanqiu_617@foxmail.com (H.Q.); 3054750963@webmail.hzau.edu.cn (Y.G.);; 2 College of Plant Science and Technology, Huazhong Agricultural University, Wuhan 430070, China; 3 College of Plant Science and Technology, Hunan Biological and Electromechanical Polytechnic, Changsha 410127, China; 4 Guizhou Livestock and Poultry Genetic Resources Management Station, Guiyang 550000, China; 5 Institute of Apicultural Research, Chinese Academy of Agricultural Sciences, Beijing 100093, China

**Keywords:** *Basilepta melanopus*, scanning electron microscopy, sensilla, morphology, sexual dimorphism

## Abstract

The tea leaf beetle *Basilepta melanopus* is a devastating leaf-feeding pest of tea plants, with its infestation area and damage severity in Chinese tea plantations continuously increasing. Characterizing the morphology and distribution of sensilla provides a morphological basis for understanding the behavioral mechanisms underlying host recognition, mate localization, and environmental perception of this pest. In this study, the morphology, ultrastructural characteristics, distribution and sexual dimorphism of sensilla on antennae, mouthparts, legs, and external genitalia of *B. melanopus* adults were examined by stereomicroscopy and scanning electron microscopy (SEM). Nine types and thirty-nine subtypes of sensilla were observed. These include various types on the antennae, including Böhm bristles, sensilla chaetica, sensilla trichodea, sensilla basiconica, sensilla auricillica, sensilla coeloconica, and sensillar pits, and many subtypes. The two types of sensilla unique to the mouthparts are sensilla styloconica and sensilla campaniformia. Sensilla chaetica and sensilla trichodea are also found on the tarsi. In addition, sensilla trichodea and sensilla basiconica were identified on the external genitalia of the female insect. On the male external genitalia, four types of sensilla were observed: sensilla trichodea, sensilla chaetica, sensilla basiconica, and sensilla coeloconica. There were also differences in the number and size of sensilla between males and females. This study investigated the relationship between the sensilla of *B. melanopus* and its key behavioral activities, namely feeding and oviposition, through detailed morphological observations and systematic analysis of these sensory structures. The findings provide a conceptual framework and offer novel perspectives for future research on the behavioral ecology and integrated management of this species.

## Simple Summary

The tea leaf beetle (*Basilepta melanopus*) is a growing threat to tea plantations in China, adults feed on tea leaves and cause serious crop losses. To develop more sustainable pest control methods, we need to understand how these beetles sense their environment—how they find tea plants, recognize mates, and choose where to feed and lay eggs. In this study, we used high-powered microscopes to examine the tiny sensory structures (sensilla) on different body parts of male and female beetles. We found a rich variety of these structures, with clear differences between the sexes. For example, males have unique smell sensors on their antennae, while females have larger touch sensors on their legs. These differences suggest that males and females use their senses differently for finding mates and selecting egg-laying sites. Our detailed map of these sensory tools provides essential clues for designing new pest management tactics, such as sex-specific traps or natural feeding repellents, that could reduce reliance on chemical pesticides and help protect tea crops more effectively.

## 1. Introduction

The tea plant (*Camellia sinensis*) is one of the most economically important beverage crops worldwide, with its cultivation history spanning thousands of years in China [1]. However, tea production is severely threatened by various insect pests, among which leaf beetles represent a particularly destructive group due to their specialized feeding habits on tea leaves [2,3]. The tea leaf beetle, *Basilepta melanopus* (Lefèvre, 1885), is a major pest species distributed across the primary tea-producing regions of China, including Hubei, Hunan, Jiangxi, Fujian, and Guangdong provinces [4]. The adults of this species feed on tea leaves, causing significant yield losses and quality degradation, which has drawn considerable attention from tea researchers and entomologists [5].

Insects rely predominantly on their sophisticated sensory systems to perceive environmental stimuli, locate host plants, identify mates, and avoid predators [6]. The sensory structures, including sensilla on the antennae, mouthparts, thoracic legs, and genitalia, constitute the peripheral nervous system that mediates olfactory, gustatory, mechanosensory, and contact chemosensory modalities [7]. Among these, the antennae serve as the primary olfactory organs, equipped with diverse sensilla types that detect volatile organic compounds released by host plants, conspecifics, and natural enemies [8,9]. The mouthparts, being the direct interface between insects and their food substrates, bear numerous sensilla responsible for gustatory perception and mechanosensory feedback during feeding [10]. The thoracic legs, particularly the tarsi, contain sensilla that contribute to substrate recognition, mate grasping, and locomotion [11]. Sensilla on the external genitalia play a crucial role in copulation behavior and reproductive isolation [12]. Additionally, binding and receptor proteins involved in volatile recognition have also been identified in the tarsi and certain other body parts [13,14].

Scanning electron microscopy (SEM) has been extensively employed to investigate the ultrastructural morphology of insect sensilla, providing high-resolution insights into their typology, distribution, and sexual dimorphism [14,15]. Previous studies on Chrysomelidae have documented various sensilla types on the antennae, mouthparts, and thoracic legs of numerous leaf beetle species, including *Leptinotarsa decemlineata* [16], *Chrysolina aeruginosa* [17], and related coleopteran species [18]. These studies have significantly advanced our understanding of the sensory basis underlying host plant selection, feeding behavior, and reproductive strategies in leaf beetles. However, the sensory morphology of *B. melanopus* remains completely unexplored, representing a significant knowledge gap in the chemical ecology and sensory physiology of this important tea pest [19].

Given the economic importance of *B. melanopus* and the critical roles of sensory structures in mediating insect–plant interactions [20]. The objectives of this study were to characterize the typology, and sexual dimorphism of sensilla on the antennae, mouthparts, thoracic legs, and external genitalia using SEM and light microscopy. We anticipate that the findings will provide a solid sensory structural basis for understanding the host location, feeding, and reproductive behaviors of this pest, and contribute to the development of novel pest management strategies targeting its sensory modalities.

## 2. Materials and Methods

### 2.1. Insect Collection

*B. melanopus* adults were collected from Jiujingfeng Tea Culture Ecological Park in Shadui Town, Tongcheng City, Hubei Province, China (29°29′ N, 113°55.8′ E). Adults were reared on fresh tea branches in an artificial climate chamber, which was maintained at (25 ± 2) °C and 60~75% relative humidity with a 12 h dark–12 h light photoperiod.

### 2.2. Observation of External Morphology

At room temperature (25 °C), under natural light conditions, adult beetles of *B. melanopus* were immobilized on Petri dishes using insect pins, and morphological characteristics of both sexes were examined under a stereomicroscope. Photomicrographs were acquired using a stereomicroscope (Leica M205 A, Leica Microsystems, Wetzlar, Germany) equipped with a CCD camera (Nikon D5100, Nikon Corporation, Tokyo, Japan) at designated magnifications.

### 2.3. Scanning Electron Microscopy (SEM) Procedures

*B. melanopus* adults (three males and three females) were selected randomly from laboratory-raised adults, and the antennae, mouthparts, legs, and external genital segments of males and females were dissected using tweezers and scalpels under a stereomicroscope (SZ61, Olympus Corporation, Tokyo, Japan). Excised parts were immediately soaked in PBS buffer solution (2.5%, Macklin Biochemical Co., Ltd., Shanghai, China) and then cleaned twice for 30 s using an ultrasonic cleaning machine (MZ101, Guangzhou Mingmei Photoelectric Technology Co., Ltd., Guangzhou, China). Each body part was then dehydrated in a graded ethanol series (70%, 80%, 90%, 95%, and 100%; each step was repeated three times for 10 min). Specimens were allowed to dry under room conditions for 15–18 min. Dried samples were then mounted on holders with conductive adhesive and sputtered with gold using a small ion sputtering instrument (JFC-1600 JEOL Ltd., Tokyo, Japan) for 30–60 s. Lastly, the prepared samples were examined using a scanning electron microscope (SU3800 Hitachi High-Technologies Corporation, Tokyo, Japan) with the acceleration voltage set at 1.0–3.0 kV. Sample preparation and observations were performed in the electron microscope room of the National Key Laboratory of Agricultural Microbiology, Huazhong Agricultural University.

### 2.4. Statistical Analysis

The naming of sensilla types was based on the classification system of Schneider [21], Zacharuk [22], Dyer and Seabrook [23], and Crook et al. [24]. The morphological terminology and classification of the sensilla used here followed Tarumingkeng [25], Zacharuk [26], and Shanbhag and Mfller [27] with regard to the type and subtypes of sensilla based on sensilla morphology, size, and distribution. The length, base diameter, quantity, and distribution of each type of sensillum from *B. melanopus* adult specimens were determined using Nano Measurer (Fudan University, Shanghai, China). All data were based on the frontal views of structures to avoid any influence from shooting angles. Images were processed with Adobe Photoshop CS6 (version 13.0; Adobe Systems Incorporated, San Jose, CA, USA). Data were analyzed with Student’s *t*-tests in GraphPad Prism version 9.0 (GraphPad Software, San Diego, CA, USA). All quantitative data are presented as the mean ± standard error (SE). An independent samples *t*-test was employed to evaluate significant differences in morphological measurements between male and female *B. melanopus* adults.

## 3. Results

### 3.1. External Morphology of B. melanopus Adults

In the adult stage, the compound eyes of *B. melanopus* are brown, and the tail of the wing was black. The adult *B. melanopus* exhibits a brown coloration. Although no significant difference in body length was observed between male and female adults (*t* = 1.428, *df* = 20, *p* = 0.169), females had a significantly wider pronotum than males (*t* = 2.697, *df* = 20, *p* = 0.014). The head and pronotum are characterized by irregularly distributed punctures, while the punctures on the elytra are arranged in rows, each encircled by a black ring. The ovipositor of the female adult is retracted within the abdominal apex, forming a distinctive “\ /”-shaped black spot (Figure 1). Data on adult body length and pronotum width are presented in Table 1.

### 3.2. Typology, Characteristics, and Distribution of Sensilla on the Antennae

The antennae of *B. melanopus* exhibit typical moniliform structures, consisting of the scape, pedicel, and flagellum (Figure 2A). The flagellum is composed of nine flagellomeres, with the distal two segments being black and the remaining segments brown. Statistical comparisons of the total antennal length and individual segment lengths between male and female adults are presented in Table 2.

A total of seven types of sensilla were identified on the antennae of both sexes, comprising eleven subtypes: Böhm bristles (BB I), sensilla chaetica (SC I, SC II), sensilla trichodea (ST I, ST II, ST III, ST IV), sensilla basiconica SB I), sensilla auricillica (Sau I), sensilla coeloconica (Sco I), and sensillar pits (SP I). Among these, SB I and Sco I were found exclusively in males. Sensilla coeloconica were observed on the 9th flagellomere segment of male antennae, characterized by a distinctly sunken basal fossa. 

The distribution and detailed morphological characteristics of these sensilla are illustrated in Figure 2 and summarized in Table 3. Sensilla dimensions and sexual dimorphism data are detailed in Table 4.

### 3.3. Typology, Characteristics, and Distribution of Sensilla on the Mouthparts

The mouthparts of *B. melanopus* are of the chewing type, consisting of the labrum, mandibles, labium, maxilla, and hypopharynx (Figure 3A). The maxilla is composed of the cardo, stipes, galea, lacinia, and maxillary palpus (Figure 3B), with sensilla predominantly distributed on the maxillae. 

Seven types of sensilla were identified on the mouthparts, comprising twelve subtypes: Böhm bristles (BB II), sensilla chaetica (SC III, SC IV), sensilla trichodea (ST V, ST VI), sensilla basiconica (SB II, SB III, SB IV), sensilla coeloconica (Sco II), sensilla styloconica (Sst I, Sst II), and sensilla campaniformia (Sca I) (Table 5, Figure 3). 

The distribution and detailed morphological characteristics of these sensilla are illustrated in Figure 3 and summarized in Table 5. Sensilla dimensions and sexual dimorphism data are detailed in Table 6.

### 3.4. Typology, Characteristics, and Distribution of Sensilla on the Legs

The thoracic legs of *B. melanopus* adults are walking legs, each composed of the coxa, trochanter, femur, tibia, tarsus, and pretarsus (Figure 4A). The distal ends of each leg segment are black, while the bases are brown, and a short spine is present in the middle of the femur. The morphologies of the prothoracic, mesothoracic, and metathoracic tarsi are consistent, belonging to the cryptopentamerous type (Figure 4B).

Two types of sensilla were identified on the thoracic legs, comprising eight subtypes: sensilla chaetica (SC V, SC VI) and sensilla trichodea (ST VII, ST VIII, ST IX, ST X, ST XI, ST XII). These sensilla are primarily distributed on the tarsi (Figure 4, Table 7).

The distribution and detailed morphological characteristics of these sensilla are illustrated in Figure 4 and summarized in Table 7. Sensilla dimensions and sexual dimorphism data are detailed in Table 8.

### 3.5. Typology, Characteristics, and Distribution of Sensilla on the External Genitalia

The types of sensilla on the external genitalia of male and female *B. melanopus* differ. The female external genitalia of *B. melanopus* are a retracted and compact structure situated at the abdominal apex. The male external genitalia exhibit a more complex and protruding architecture compared to those of the female, with the structure dominated by the phallobase, which serves as the primary copulatory organ.

On the external genitalia of females, two types of sensilla were identified: sensilla trichodea (ST XIII, ST XIV) and sensilla basiconica (SB V, SB VI). On the external genitalia of males, four types of sensilla were observed: sensilla trichodea (ST XV), sensilla chaetica (SC VII), sensilla basiconica (SB VII), and sensilla coeloconica (Sco III) (Figure 5, Table 9).

The distribution and detailed morphological characteristics of these sensilla are illustrated in Figure 5 and summarized in Table 9. Sensilla dimensions are detailed in Table 9.

## 4. Discussion

### 4.1. Antennal Sensilla and Olfactory Ecology

The antennae of *B. melanopus* adults exhibit a moniliform–filiform structure with 11 segments, consistent with the generalized antenna plan of Chrysomelidae [18]. The significant sexual dimorphism in antennal length, with males bearing longer flagellomeres (particularly F1, F4, F5, F8, and F9), suggests the possibility of differences in olfactory capacity, but further verification is needed [28]. Such dimorphism is widespread in Coleoptera and has been linked to the distinct reproductive roles of males (active mate searching) and females (passive attraction and oviposition site selection) [16].

Seven types of antennal sensilla were identified in males, compared to five types in females, with SB I and Sco I being male-specific. The absence of SB I in females is noteworthy, as sensilla basiconica are generally considered bimodal chemo-mechanoreceptors involved in the detection of host plant volatiles and contact chemoreception [7]. The exclusive presence of Sco I on the ninth flagellomere of males suggests a specialized role in the perception of sex pheromones or male-specific kairomones, as coeloconic sensilla are known to house olfactory receptor neurons tuned to specific volatile classes [29]. The significant sexual differences in the length of BB I, ST I, and Sau I, as well as the basal width of ST II, ST IV, and SP I, further suggest possible functional divergence of antennal sensory equipment between sexes [18].

The distribution patterns of antennal sensilla provide additional clues to their functional roles. SC I, concentrated at the proximal end of each flagellomere, likely serve mechanosensory and proprioceptive functions, monitoring antennal movement and position [11]. While sensilla trichodea are widely distributed across all flagellomeres, their subtypes show distinct distribution patterns: ST III occurs throughout F1–F9, whereas ST I and ST II are concentrated on the distal flagellomeres (F5–F9). This differential distribution suggests their involvement in olfactory detection, with the concentration of ST I–II on F5–F9 aligning with the active waving of these distal segments during odor sampling behavior [28]. SP I, distributed from F3 to F9, may function as hygroreceptors or thermoreceptors, contributing to microclimate assessment within the tea canopy [5]. Nevertheless, functional validation remains to be performed.

### 4.2. Mouthpart Sensilla and Feeding Behavior

The chewing mouthparts of *B. melanopus*, comprising the labrum, mandibles, labium, maxillae, and hypopharynx, bear a diverse array of seven sensilla types arranged in twelve subtypes [30]. The concentration of sensilla on the maxillae, particularly the maxillary palpi, aligns with the critical role of these structures in food evaluation and manipulation prior to ingestion [10]. SC III on the maxillary and labial palpi, characterized by their smooth surface and large angle with the cuticle, are typical mechanoreceptors that provide tactile feedback during palpation of leaf surfaces.

The presence of sensilla styloconica at the apices of the labial and maxillary palpi is particularly significant, as these sensilla are generally considered gustatory in many Coleoptera [7]. The morphological differentiation between Sst I (with a longitudinal apical groove) and Sst II (with a globular apical protrusion) may reflect functional partitioning in the detection of phagostimulants (e.g., sugars, amino acids) and deterrents (e.g., alkaloids, tannins) present in tea leaves [6]. The small SB II at the palpal apices, with their characteristic apical pores, are likely contact chemoreceptors involved in the final acceptance or rejection of food substrates [7].

The distribution of Sco II on the inner surface of the labrum and Sca I on the maxillary palpus suggests additional mechanosensory roles in monitoring labrum movement and detecting cuticular strain during feeding [10]. The large SC IV on the dorsal labrum, with its prominent basal depression, may function as defensive mechanoreceptors triggering protective reflexes when the mouthparts encounter physical obstacles [11]. Nevertheless, functional validation remains to be performed.

### 4.3. Thoracic Leg Sensilla and Locomotory Functions

The thoracic legs of *B. melanopus*, typical of walking legs in Chrysomelidae, bear two principal sensilla types (chaetica and trichodea) differentiated into eight subtypes. The predominance of sensilla on the tarsi, particularly the ventral surfaces of tarsomeres, reflects the critical importance of tarsal chemoreception in substrate recognition and host plant acceptance [12]. The brush-like arrangement of SC VI (13–15 count) at the distal tibia is characteristic of tibial mechanoreceptor arrays that detect ground contact and proprioceptive signals during walking.

The remarkable sexual dimorphism in thoracic leg sensilla, particularly the striking differences in ST VIII, ST IX, ST X, and ST XI, suggests possible sex-specific roles that require functional validation. The significantly longer and wider ST VIII in females may enhance mechanosensory sensitivity for oviposition substrate evaluation, as females must assess leaf toughness, surface chemistry, and moisture content prior to egg deposition. Conversely, the longer ST X in males, restricted to the foreleg and midleg tarsomeres, could be related to male-specific behaviors, such as mate grasping, but this remains speculative [16]. The male-specific ST XII on the hindleg tarsus, with its distinctive golf club-shaped apex, represents a novel sensillum subtype that warrants further electrophysiological investigation to elucidate its precise function [17].

The cryptopentamerous tarsal structure, consistent across all thoracic legs, is a derived condition within Chrysomelidae and has been associated with enhanced tarsal flexibility and adhesion on smooth leaf surfaces [31]. The sensilla complement on the tarsi may contribute to substrate discrimination, but host cultivar preference remains to be tested [32]. Nevertheless, functional validation remains to be performed.

### 4.4. Genital Sensilla and Reproductive Behavior

The marked sexual divergence in genital sensilla types—females bearing two types (ST XIII, ST XIV, SB V, SB VI) versus males bearing four types (ST XV, SC VII, SB VII, Sco III)—reflects the distinct functional demands of copulatory organs in each sex [12]. The elongated ST XV on the male phallobase may be involved in mechanoreception during copulation [16]. The Sco III positioned within the phallobase groove could have a chemosensory function, but this requires investigation [6].

On the female pseudoovipositor, the SB V with their raised bases and rounded apices, together with the sharper SB VI, probably function in mechanosensory and chemosensory evaluation of oviposition substrates [12]. The reduced sensilla complement in female genitalia compared to males may reflect different sensory requirements between sexes, but the exact behavioral roles have yet to be determined [16]. Nevertheless, functional validation remains to be performed.

### 4.5. Evolutionary Implications of Sensillar Variation

To further contextualize the evolutionary patterns of sensillar morphology within the Chrysomelidae, we extended our comparative analysis to include *Diabrotica virgifera* (western corn rootworm), a well-studied species within the same family [33]. Similar to *B. melanopus* and *A. hygrophila*, *D. virgifera* possesses the core sensillar types commonly found in chrysomelids, including sensilla chaetica, trichodea, and basiconica. However, marked differences emerge at the subtype and distribution levels. In *D. virgifera*, the longer male antennae bear more sensilla overall, and the terminal antennal segment of males possesses six sensillar types not found on the corresponding female segment, while females possess four types unique to their terminal segment. This pattern of sexual dimorphism—where both sexes possess sex-specific sensilla—contrasts with the male-specific SB I and Sco I observed exclusively in *B. melanopus*, and the female-specific subtypes and petal-shaped sensillum (Sps) documented in *A. hygrophila* [34]. The divergent expression of sexual dimorphism across these three chrysomelid species suggests that while the family-level sensillar ground plan is highly conserved, the specific patterns of sex-specific sensory adaptations are lineage-dependent, likely shaped by differences in reproductive behavior, host plant associations, and ecological niches. These comparisons reinforce the view that core sensillar types represent ancestral conditions for the Chrysomelidae, whereas subtype diversity, sexual dimorphism patterns, and unique sensilla reflect species-specific adaptive evolution.

### 4.6. Implications for Pest Management

The comprehensive sensory morphological data generated in this study have direct implications for the development of behavior-based pest management strategies for *B. melanopus* [2]. The identification of olfactory sensilla types (ST I–IV, SB I, Sco I) on the antennae provides targets for the screening of behaviorally active compounds, including host plant volatiles, aggregation pheromones, and sex pheromones [7]. The gustatory sensilla on the mouthparts (Sst I, Sst II, SB II) and tarsi (SB II analogues) offer opportunities for the development of antifeedants and feeding deterrents that disrupt host plant recognition [35].

The sexual dimorphism in sensory equipment, particularly the male-specific antennal sensilla (SB I, Sco I) and the female-enlarged leg sensilla (ST VIII), suggests that sex-specific lures or traps could be optimized by targeting the distinct chemosensory preferences of each sex [28]. Furthermore, the geographic variation in elytral patterns highlights the need for region-specific monitoring protocols and the potential for population-level management strategies in different tea-producing areas [36].

These findings resonate with successful sensory-based management strategies in other beetles. For example, pheromone-based trapping has been widely applied in Cerambycidae, with pheromones now identified from more than 100 species, and optimized traps and lures have been developed for detection, sampling, and management [37]. In Scolytinae, push–pull semiochemical strategies have been shown to significantly reduce populations of Dendroctonus and Ips [38]. In Chrysomelidae, tarsal gustatory sensilla respond electrophysiologically to salts, sugars, bitter substances, and host leaf surface waxes, providing a basis for feeding deterrent development [39]. However, the specific sensillum assemblages and sexual dimorphism patterns in *B. melanopus* are distinct from those in Cerambycidae or Scolytinae, where pheromone structures are often conserved across species. Moreover, the geographic variation documented here is morphological (elytral patterns), whereas in other beetles, geographic variation in pheromone systems—such as the differential attraction responses among California, Idaho, and New York populations of *Ips pini*—has been well documented [40]. This contrast emphasizes that despite the shared conceptual framework, species-specific and region-tailored approaches are essential.

## 5. Conclusions

This study presents a detailed investigation of the sensillar ultrastructure in *B. melanopus*. We documented and characterized the typology and sexual dimorphism of sensilla on the antennae, mouthparts, thoracic legs, and external genitalia. The findings establish a comprehensive sensory structural foundation for understanding the behavioral ecology of this important tea pest. Future research can employ transmission electron microscopy to investigate the internal structures of various sensilla, utilize electrophysiological techniques to analyze their functions, and thereby develop targeted pheromone traps to enhance integrated control strategies for *B. melanopus*.

## Figures and Tables

**Figure 1:**
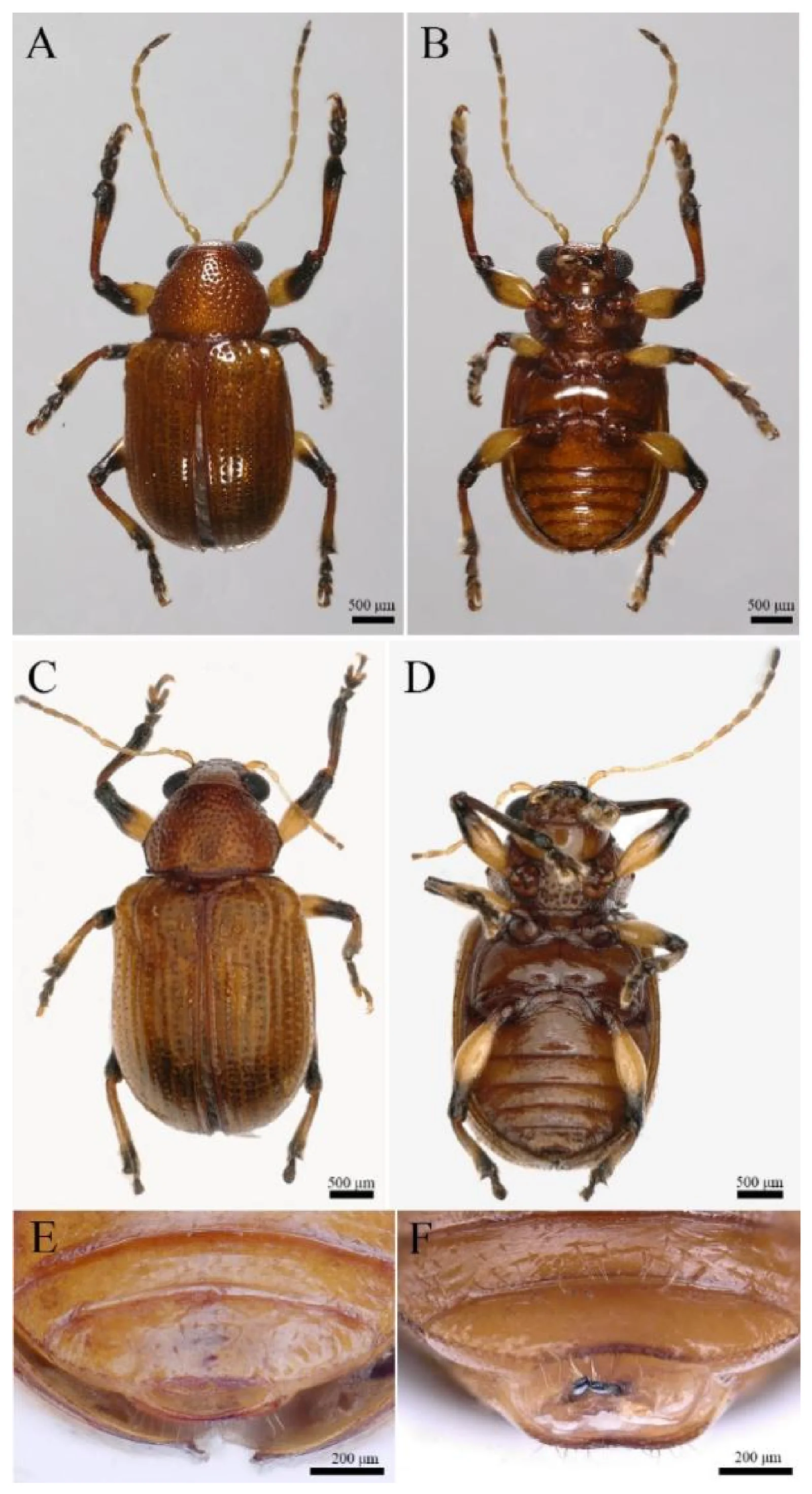
External morphology of *B. melanopus* adults: (**A**) Dorsal view of male adult; external morphology of *B. melanopus* adult. (**B**) Ventral view of male adult. (**C**) Dorsal view of female adult. (**D**) Ventral view of female adult. (**E**) Abdominal end of male. (**F**) Abdominal end of female with an “\ /”-shaped black spot.

**Figure 2:**
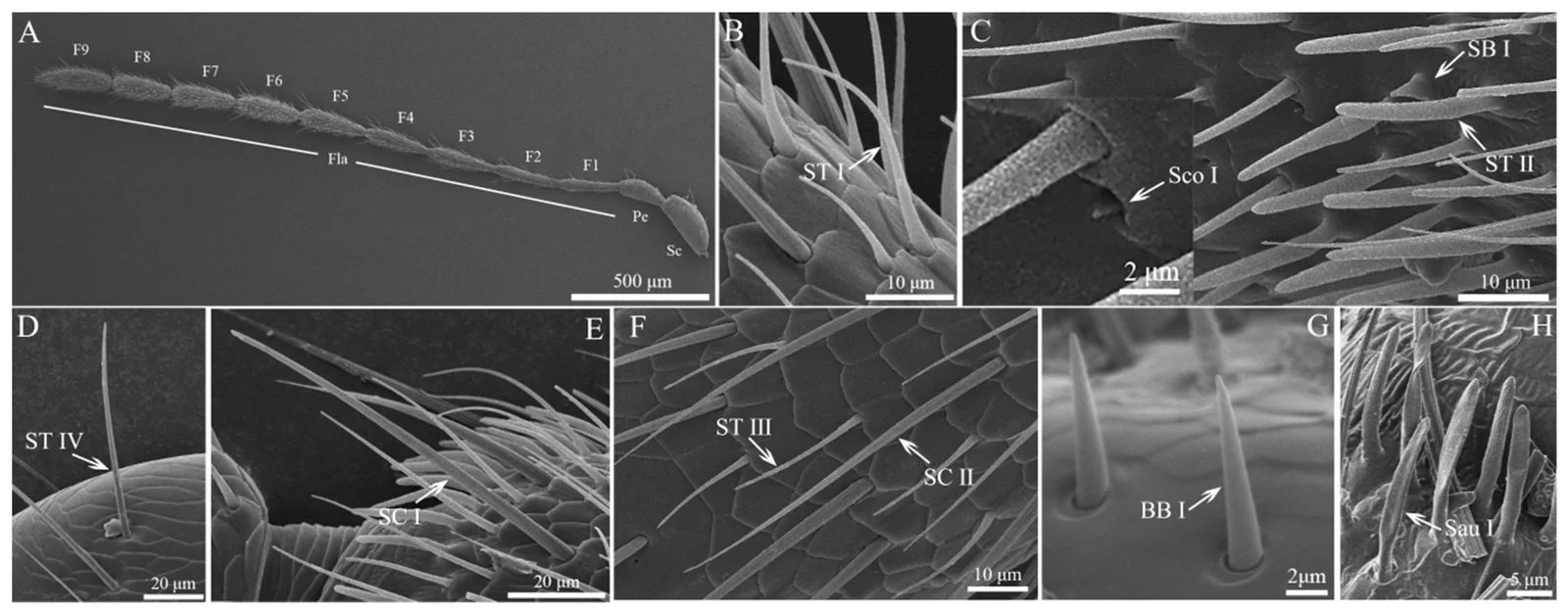
(**A**) The whole antenna. Sc, scape, Pe, pedicle, Fla, flagellum. SEM structure of sensilla on antennae of *B. melanopus*. (**B**) Sensilla trichodea (ST I). (**C**) Sensilla trichodea (ST II), Sensilla coeloconica (Sco I), Sensilla basiconicum (SB I). (**D**) Sensilla trichodea (ST IV). (**E**) Sensilla chaetica (SC I). (**F**) Sensilla trichodea (ST III), Sensilla chaetica (SC II). (**G**) Böhm Bristles (BB I). (**H**) Sensilla auricillica (Sau I).

**Figure 3:**
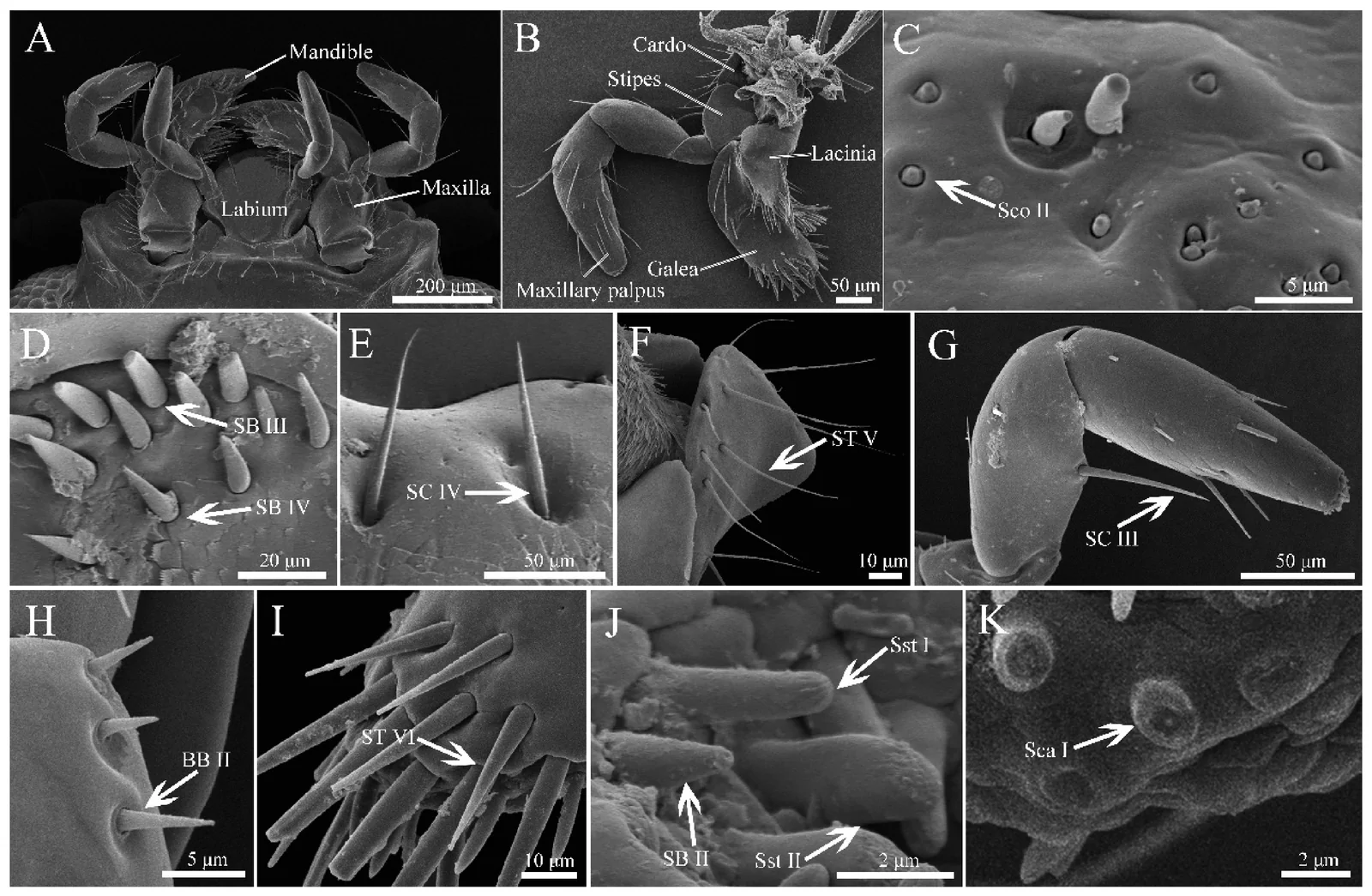
Scanning electron micrographs (SEMs) of the mouthparts and sensilla of adult *B. melanopus*: (**A**) overall ventral view of the mouthparts; (**B**) maxilla showing anatomical structures; (**C**) sensilla coeloconica (Sco II); (**D**) sensilla basiconica (SB III, SB IV); (**E**) sensilla chaetica (SC IV); (**F**) sensilla trichodea (ST V); (**G**) sensilla chaetica (SC III); (**H**) Böhm bristles (BB II); (**I**) sensilla trichodea (ST VI); (**J**) sensilla styloconica (Sst I, Sst II) and sensilla basiconica (SB II); (**K**) sensilla campaniformia (Sca I).

**Figure 4:**
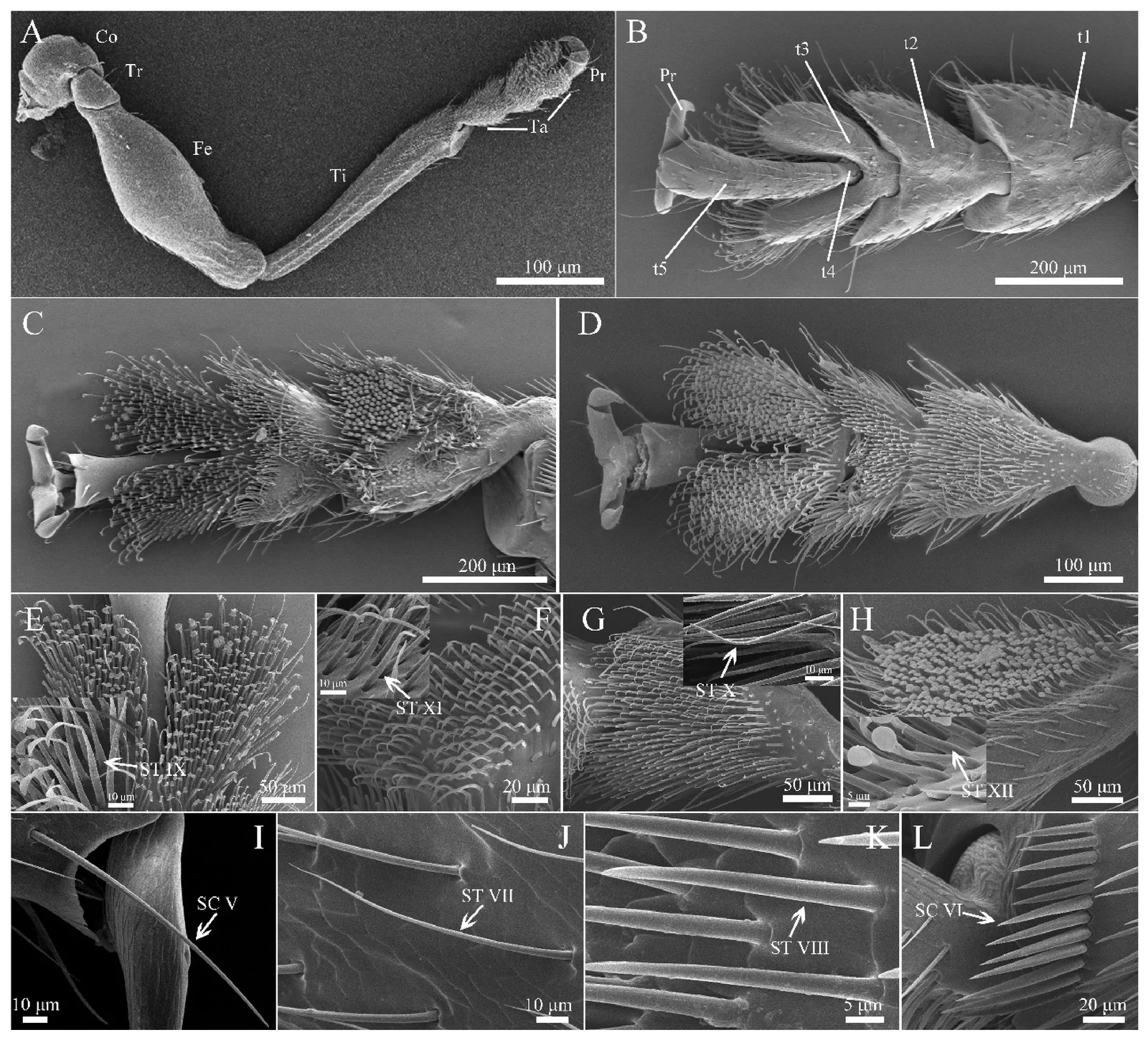
Scanning electron micrographs (SEMs) of the thoracic legs and leg sensilla of adult *B. melanopus*: (**A**) The foreleg of *B. melanopus* female adult. Co, coxa; Tr, trochanter; Fe, femur; Ti, tibia; Ta, tarsus; Pr, pretarsus. SEM structure of sensilla on leg of *B. melanopus*. (**B**) Dorsal view of tarsus of thoracic legs. Pr, pretarsus. (C) Ventral view of protarsus of *B. melanopus* male adult. (**D**) Ventral view of protarsus of *B. melanopus* female adult. (**E**) Sensilla trichodea (ST IX). (**F**) Sensilla trichodea (ST XI). (**G**) Sensilla trichodea (ST X). (**H**) Sensilla trichodea (ST XII). (**I**) Sensilla chaetica (SC V). (**J**) Sensilla trichodea (ST VII). (**K**) Sensilla trichodea (ST III). (**L**) Sensilla chaetica (SC VI).

**Figure 5:**
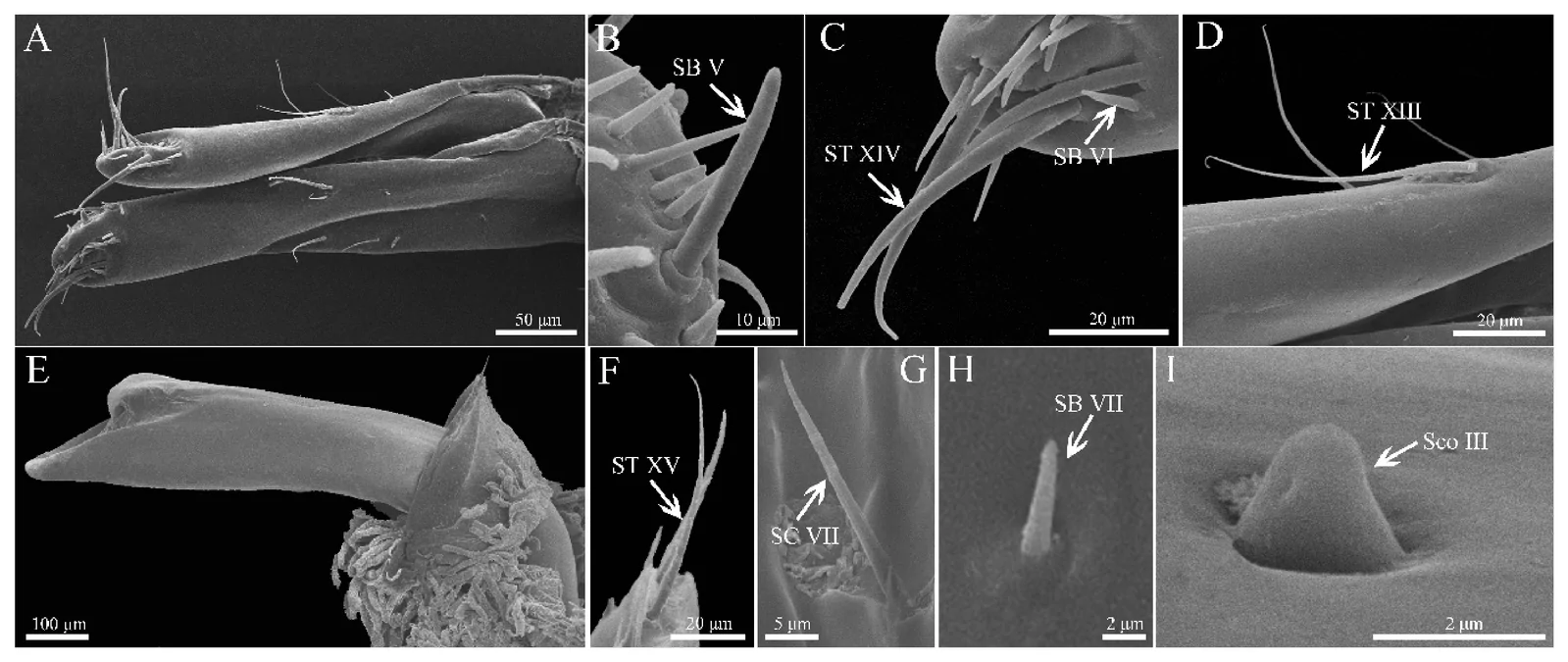
Scanning electron micrographs (SEMs) of external genitalia and genital sensilla of *B. melanopus* adults: (**A**) Overall view of female external genitalia. SEM structure of sensilla on genital segments of *B. melanopus*. (**B**) Sensilla basiconicum (SB V). (**C**) Sensilla trichodea (ST XIV), Sensilla basiconicum (SB VI). (**D**) Sensilla trichodea (ST XIII). (**E**) Overall view of male external genitalia. (**F**) Sensilla styloconica (ST XV). (**G**) Sensilla chaetica (SC VII). (**H**) Sensilla basiconicum (SB VII). (**I**) Sensilla coeloconica (Sco III).

**Table 1:** Mean length and pronotum width of *B. melanopus* adults.

Life Stage	Gender	Sample Size (*n*)	Pronotum Width (mm)	Length (mm)
Adult	Male	11	1.405 ± 0.024	3.610 ± 0.075
	Female	11	1.524 ± 0.037 *	3.788 ± 0.100

Note: Differences between male and female adults were analyzed using an independent samples *t*-test (“*”, *p* < 0.05).

**Table 2:** Mean length and width of antennal segments in female and male *B. melanopus* adults.

Antenna Segments		Length (μm)		Statistical Analysis	Width (μm)		Statistical Analysis
		Male	Female		Male	Female	
Scape		235.71 ± 3.29	239.80 ± 7.83	*t* = 0.3386, *df* = 9.509, *p* = 0.9737	106.46 ± 6.21	100.24 ± 3.61	*t* = 0.8648, *df* = 8.033, *p* = 0.4122
Pedicel		210.14 ± 0.53 *	191.90 ± 5.80	*t* = 3.131, *df* = 5.082, *p* = 0.025	75.04 ± 3.52	74.26 ± 2.35	*t* = 0.1852, *df* = 8.708, *p* = 0.8573
Flagellum	F1	258.92 ± 2.19 **	222.24 ± 7.59	*t* = 4.643, *df* = 5.828, *p* = 0.004	55.09 ± 2.32	50.56 ± 1.67	*t* = 1.585, *df* = 9.089, *p* = 0.1472
	F2	275.07 ± 3.81 *	253.22 ± 8.93	*t* = 2.250, *df* = 10, *p* = 0.048	58.56 ± 1.89	54.41 ± 1.97	*t* = 1.522, *df* = 9.984, *p* = 0.1590
	F3	297.83 ± 10.46	278.10 ± 7.38	*t* = 1.575, *df* = 5.931, *p* = 0.1670	68.84 ± 3.62	63.02 ± 2.42	*t* = 1.337, *df* = 8.716, *p* = 0.2150
	F4	285.44 ± 4.78 **	249.80 ± 6.97	*t* = 4.218, *df* = 10, *p* = 0.002	70.45 ± 5.18	68.44 ± 2.27	*t* = 0.3548, *df* = 6.857, *p* = 0.7334
	F5	305.17 ± 2.18 *	274.41 ± 8.73	*t* = 3.420, *df* = 5.622, *p* = 0.016	79.57 ± 5.52	76.68 ± 3.22	*t* = 0.4515, *df* = 8.060, *p* = 0.6635
	F6	264.98 ± 4.00	258.66 ± 8.95	*t* = 2.245, *df* = 9.517, *p* = 0.05	76.04 ± 3.71	75.97 ± 2.63	*t* = 0.01540, *df* = 9.011, *p* = 0.9880
	F7	269.98 ± 5.23	250.98 ± 8.35	*t* = 1.404, *df* = 8.050, *p* = 0.1978	80.10 ± 3.70	76.21 ± 3.05	*t* = 0.8104, *df* = 9.643, *p* = 0.4373
	F8	271.88 ± 1.98 **	233.50 ± 6.83	*t* = 5.397, *df* = 5.830, *p* = 0.002	83.74 ± 2.92	76.87 ± 2.76	*t* = 1.706, *df* = 9.969, *p* = 0.1189
	F9	333.20 ± 2.98 **	275.39 ± 11.41	*t* = 4.903, *df* = 5.677, *p* = 0.003	86.08 ± 3.56	79.11 ± 2.98	*t* = 1.500, *df* = 9.697, *p* = 0.1655
Total length		3008.32 ± 30.23 **	2728.00 ± 69.16	*t* = 3.714, *df* = 6.844, *p* = 0.008	N/A	N/A	N/A

Note: Data are presented as mean ± SE (*n* = 6). Differences between male and female adults are analyzed using Welch’s *t*-test to account for unequal variances (* *p* < 0.05, ** *p* < 0.01). N/A = not applicable.

**Table 3:** Distribution and morphological characteristics of antennal sensilla in *B. melanopus*.

Sensilla Subtype	Distribution	Morphological Characteristics
BB I	the junction of the pedicel and scape	Böhm bristles I (BB I) possess an obvious basal fossa and a smooth surface. These bristles taper from the base to a sharp apex and are oriented perpendicularly to the antennal surface (Figure 2G).
SC I	the proximal end of each segment of flagellum	Sensilla chaetica I (SC I) are characterized by a distinct basal fossa and longitudinal grooves on their surface, gradually tapering from the base to a sharp tip, and are straight (Figure 2E).
SC II	F3–F9	Sensilla chaetica II (SC II) are characterized by a distinct basal fossa and longitudinal grooves on their surface, gradually tapering from the base to a sharp tip. SC II exhibits a slight curvature towards the antenna at its apex and is thinner and shorter than SC I (Figure 2F).
ST I	F5–F9	Sensilla trichodea I (ST I) are distributed on parts of the antennal segments, featuring a shallow basal fossa, a smooth surface, and a gradual taper from base to apex. ST I originates distally on the antennae, possesses a sharp apex, and is distinctly curved towards the antennal side (Figure 2B).
ST II	F5–F9	Sensilla trichodea II (ST II) are distributed on parts of the antennal segments, featuring a shallow basal fossa, a smooth surface, and a gradual taper from base to apex. ST II represents the shortest sensilla trichodea on the antennae, with a rounded and blunt apex (Figure 2C).
ST III	F1–F9	Sensilla trichodea III (ST III) are distributed on parts of the antennal segments, featuring a shallow basal fossa, a smooth surface, and a gradual taper from base to apex. ST III is slender at the base, with a sharp apex, and is positioned closer to the antennal surface (Figure 2F).
ST IV	scape, pedicle	Sensilla trichodea IV (ST IV) are distributed on parts of the antennal segments, featuring a shallow basal fossa, a smooth surface, and a gradual taper from base to apex. ST IV constitutes the longest sensilla trichodea on the antennae, having a sharp apex and oriented almost perpendicularly to the antennal surface (Figure 2D).
SB I	F7–F9	Sensilla basiconica I (SB I) are short, with a prominent basal fossa, a smooth surface, and a blunt apex (Figure 2C).
Sau I	F6–F8	Sensilla auricillica I (Sau I) possess a smooth surface and form a small angle with the antennal surface, lacking an obvious basal fossa. Their external morphology resembles the rolled leaf-like structure characteristic of Poaceae plants, featuring ear-like grooves and a rounded, blunt apex (Figure 2H).
Sco I	F9	Sensilla coeloconica I (Sco I) were characterized by a distinctly sunken basal fossa.
SP I	F3–F9	Sensilla pits I (SP I) appeared as downwardly concave, rounded holes.

**Table 4:** Main sensilla differences in antennae between male and female *B. melanopus* adults.

Sensilla Subtype	Gender	Sample Size (*n*)	Length (µm)	Statistical Analysis	Basal Width (µm)	Statistical Analysis
BB I	Female	15	6.40 ± 0.46	*t* = 3.142, *df* = 28, *p* = 0.004	1.69 ± 0.10	*t* = 1.229, *df* = 57.46, *p* = 0.2239
	Male	15	8.43 ± 0.45 *		1.77 ± 0.06	
SC I	Female	15	69.42 ± 3.02	*t* = 1.937, *df* = 28.05, *p* = 0.0629	3.64 ± 0.19	*t* = 1.781, *df* = 33.63, *p* = 0.0840
	Male	15	78.54 ± 4.51		4.01 ± 0.19	
SC II	Female	15	53.18 ± 1.85	*t* = 1.644, *df* = 28.57, *p* = 0.1112	2.54 ± 0.11	*t* = 0.9167, *df* = 25.94, *p* = 0.3677
	Male	15	50.88 ± 2.21		2.70 ± 0.13	
ST I	Female	15	34.12 ± 0.89	*t* = 2.760, *df* = 28, *p* = 0.010	2.28 ± 0.05	*t* = 0.5527, *df* = 26.17, *p* = 0.5851
	Male	15	37.73 ± 0.96 *		2.16 ± 0.06	
ST II	Female	15	15.37 ± 0.49	*t* = 1.722, *df* = 31.67, *p* = 0.0949	2.03 ± 0.06	*t* = 2.633, *df* = 28, *p* = 0.014
	Male	15	16.87 ± 0.93		2.34 ± 0.10 *	
ST III	Female	15	23.35 ± 1.18 *	*t* = 2.342, *df* = 43.73, *p* = 0.0238	1.90 ± 0.05	*t* = 1.472, *df* = 44.37, *p* = 0.1482
	Male	15	21.63 ± 0.88		1.79 ± 0.05	
ST IV	Female	8	53.55 ± 4.42	*t* = 1.199, *df* = 18.21, *p* = 0.2459	2.42 ± 0.25	*t* = 3.096, *df* = 14, *p* = 0.008
	Male	8	63.82 ± 6.99		3.58 ± 0.27 *	
SB I	Female	N/A	N/A	N/A	N/A	N/A
	Male	5	4.24 ± 0.21	N/A	2.98 ± 0.11	N/A
Sau I	Female	10	18.40 ± 0.43	*t* = 5.990, *df* = 18, *p* < 0.001	2.33 ± 0.10	*t* = 1.947, *df* = 17.99, *p* = 0.0673
	Male	10	22.22 ± 0.45 *		2.54 ± 0.11	
Sco I	Female	N/A	N/A	N/A	N/A	N/A
	Male	1	1.36	N/A	1.00	N/A
SP I	Female	12	N/A	N/A	0.80 ± 0.03	*t* = 3.083, *df* = 22, *p* = 0.005
	Male	12	N/A	N/A	0.68 ± 0.02 *	

Note: Data are presented as mean ± SE. Differences between male and female adults are analyzed using an independent samples *t*-test. N/A = not available. An asterisk (*) indicates a significant difference between sexes (*p* < 0.05). Sco I is observed in only one male specimen (*n* = 1), so standard error cannot be calculated.

**Table 5:** Distribution and morphological characteristics of mouthpart sensilla in *B. melanopus*.

Sensilla Subtype	Distribution	Morphological Characteristics
BB II	maxilla	BB II exhibited an obvious basal fossa and was oriented almost perpendicularly to the surface of the maxillary palpus (Figure 3H).
ST V	mandible, maxilla	Sensilla trichodea V (ST V) feature a shallow basal fossa, a smooth surface, and a gradual taper from base to apex.
ST VI	maxilla	Sensilla trichodea VI (ST VI) feature a shallow basal fossa, a smooth surface, and a gradual taper from base to apex.
SC III	labium, maxilla	SC III is characterized by a smooth surface and forms a large angle with the mouthpart surface (Figure 3G).
SC IV	labrum, maxilla	SC IV forms a small angle with the labrum (Figure 3E).
SB II	labium, maxilla	SB II features sharp ends and small apical pores (Figure 3G).
SB III	labrum	SB III has a circular cross-section at its tip (Figure 3D).
SB IV	labrum	SB IV is characterized by possessing a sharp apex (Figure 3D).
Sst I	maxilla, labium	Sst I exhibits a longitudinal groove at the end of the sensillum (Figure 3J).
Sst II	maxilla, labium	Sst II possesses a globular protrusion at the end of the sensillum (Figure 3J).
Sco II	labrum	The base of Sco II is distinctly sunken downwards and cone-shaped (Figure 3C).
Sca I	maxilla	Sensilla campaniformia are characterized by a hemispherical protrusion on the body wall (Figure 3K).

**Table 6:** External morphology of mouthpart sensilla in adult *B. Melanoplus*.

Sensilla Subtype	Sample Size (*n*)	Length (µm)	Basal Width (µm)
BB II	4	6.15 ± 1.39	1.44 ± 0.13
ST V	6	36.41 ± 3.00	1.79 ± 0.10
ST VI	16	36.61 ± 2.22	4.79 ± 0.20
SC III	18	51.47 ± 3.57	3.64 ± 0.29
SC IV	8	84.80 ± 3.43	6.14 ± 0.32
SB II	12	3.48 ± 0.48	1.29 ± 0.12
SB III	11	14.24 ± 0.50	6.31 ± 0.24
SB IV	6	17.53 ± 0.42	6.37 ± 0.26
Sst I	14	2.68 ± 0.12	0.98 ± 0.03
Sst II	15	3.20 ± 0.09	1.70 ± 0.04
Sco II	9	N/A	1.34 ± 0.03
Sca I	7	N/A	1.78 ± 0.06

Note: Data are presented as mean ± SE (*n* represents the sample size). N/A = not applicable (length cannot be measured due to sunken or dome-like structure).

**Table 7:** Distribution and morphological characteristics of leg sensilla in *B. melanopus*.

Sensilla Subtype	Distribution	Morphological Characteristics
SC V	Tarsus	SC V forms a large angle with the tarsal surface (Figure 4I).
SC VI	Tibia	SC VI is characterized by a distinct basal pit and vertical striations on its surface; 13–15 SC VI sensilla are arranged in a brush-like pattern at the tibial apex (Figure 4L).
ST VII	Coxa, trochanter, femur	ST VII has a minor presence on the coxa and trochanter. It possesses an obvious basal fossa and a curved apex oriented towards the leg surface (Figure 4J).
ST VIII	Tibia, tarsus	ST VIII has a shallow basal fossa (Figure 4K).
ST IX	Tarsomere 3	ST IX has a flattened base, a golf-club-shaped apex, and longitudinal stripes on its surface (Figure 4E).
ST X	Tarsomere 1	ST X features an obvious basal fossa and a sharp apex, slightly curved towards the ventral surface of the tarsomere (Figure 4G).
ST XI	Tarsomere 2	ST XI has a flat apex bent at nearly 90° (Figure 4F).
ST XII	Tarsomere 1	ST XII is characterized by a slightly curved middle section and an enlarged, circular cross-section at the apex, with vertical stripes on its surface (Figure 4H).

**Table 8:** Main sensilla differences in legs between male and female *B. melanopus* adults.

Sensilla Subtype	Gender	Sample Size (*n*)	Length (µm)	Statistical Analysis	Basal Width (µm)	Statistical Analysis
SC V	Female	20	102.40 ± 1.23	*t* = 1.580, *df* = 36.97, *p* = 0.1226	3.92 ± 0.09	*t* = 0.9074, *df* = 36.58, *p* = 0.3701
	Male	20	105.24 ± 2.15		3.80 ± 0.10	
SC VI	Female	50	50.25 ± 0.55 ***	*t* = 3.916, *df* = 84.342, *p* < 0.001	6.62 ± 0.12	*t* = 2.551, *df* = 86.72, *p* = 0.1125
	Male	50	46.30 ± 0.84		6.47 ± 0.10	
ST VII	Female	15	83.04 ± 3.44	*t* = 11.13, *df* = 22.07, *p* = 0.1723	3.38 ± 0.13	*t* = 2.072, *df* = 33.41, *p* = 0.1460
	Male	15	90.89 ± 4.14		3.09 ± 0.11	
ST VIII	Female	25	91.18 ± 3.35 ***	*t* = 9.153, *df* = 48, *p* < 0.001	5.28 ± 0.16 ***	*t* = 3.753, *df* = 48, *p* < 0.001
	Male	25	48.63 ± 3.22		4.43 ± 0.16	
ST IX	Female	15	77.97 ± 1.61 ***	*t* = 4.507, *df* = 18.777, *p* < 0.001	3.07 ± 0.10	*t* = 2.504, *df* = 29.53, *p* = 0.1180
	Male	15	59.17 ± 3.85		3.21 ± 0.16	
ST X	Female	15	49.30 ± 2.99	*t* = 5.024, *df* = 19.494, *p* < 0.001	3.20 ± 0.15	*t* = 0.4541, *df* = 25.66, *p* = 0.6535
	Male	15	65.78 ± 1.35 ***		3.12 ± 0.08	
ST XI	Female	13	58.06 ± 2.92 **	*t* = 3.430, *df* = 24, *p* = 0.002	3.52 ± 0.12 ***	*t* = 5.682, *df* = 14.238, *p* < 0.001
	Male	13	44.95 ± 2.46		2.79 ± 0.04	
ST XII	Female	-	N/A	N/A	N/A	N/A
	Male	20	31.90 ± 1.43	N/A	3.10 ± 0.12	N/A

Note: Data in the table are presented as mean ± SE. Differences between male and female adults are analyzed using an independent samples *t*-test. N/A = not applicable. For the same parameter in a row, asterisks indicates there is a significant difference between male and female adults. “**” indicates *p* < 0.01, “***” indicates *p* < 0.001.

**Table 9:** Morphological characteristics and dimensions of external genitalia sensilla in female and male *B. melanopus* adults.

Sensilla Subtype	Gender	Sample Size (*n*)	Length (µm)	Basal Width (µm)
ST XIII	Female	3	36.93 ± 13.50 (15.97, 32.66, 62.16)	2.35 ± 0.25 (1.87, 2.73, 2.45)
ST XIV		8	57.64 ± 2.67	4.08 ± 0.15
SB V		3	47.12 ± 7.83 (56.03, 31.52, 53.82)	5.10 ± 1.65 (8.40, 3.25, 3.66)
SB VI		13	18.78 ± 1.93	3.38 ± 0.44
ST XV	Male	2	59.19 ± 8.56 (67.75, 50.63)	3.06 ± 0.18 (3.24, 2.88)
SC VII		3	30.27 ± 4.78 (25.41, 39.82, 25.59)	2.46 ± 0.06 (2.52, 2.52, 2.34)
SB VII		4	5.91 ± 0.23	1.67 ± 0.06
Sco III		17	N/A	2.19 ± 0.04

Note: Data are presented as mean ± SE (*n* represents the sample size). N/A = not applicable (length is not measured due to sunken pit structure). For groups with *n* = 2 and *n* = 3, the sample sizes are below the threshold for robust variance estimation. For detailed data, please refer to the table. These particular results should be viewed with caution and are not suitable for generalization.

## Data Availability

The original contributions presented in this study are included in the article. Further inquiries can be directed to the corresponding authors.
